# Turnover of Variant Surface Glycoprotein in Trypanosoma brucei Is Not Altered in Response to Specific Silencing

**DOI:** 10.1128/msphere.00122-22

**Published:** 2022-06-21

**Authors:** Mohamed Sharif, Paige Garrison, Peter Bush, James D. Bangs

**Affiliations:** a Department of Microbiology and Immunology, Jacobs School of Medicine and Biomedical Sciences, University at Buffalo (SUNY), Buffalo, New York, USA; b South Campus Instrument Center, School of Dental Medicine, University at Buffalo (SUNY), Buffalo, New York, USA; University of Texas Southwestern

**Keywords:** trypanosome, variant surface glycoprotein, RNAi silencing, glycosylphosphatidylinositol, protein turnover

## Abstract

African trypanosomes evade the immune system of the mammalian host by the antigenic variation of the predominant glycosylphosphatidylinositol (GPI)-anchored surface protein, variant surface glycoprotein (VSG). VSG is a very stable protein that is turned over from the cell surface with a long half-life (~26 h), allowing newly synthesized VSG to populate the surface. We have recently demonstrated that VSG turnover under normal growth is mediated by a combination of GPI hydrolysis and direct shedding with intact GPI anchors. VSG synthesis is tightly regulated in dividing trypanosomes, and when subjected to RNA interference (RNAi) silencing, cells display rapid cell cycle arrest in order to conserve VSG density on the cell surface (K. Sheader, S. Vaughan, J. Minchin, K. Hughes, et al., Proc Natl Acad Sci U S A 102:8716–8721, 2005, https://doi.org/10.1073/pnas.0501886102). Arrested cells also display an altered morphology of secretory organelles—engorgement of the *trans*-Golgi cisternae—that may reflect a disruption of post-Golgi secretory transport. We now ask whether trypanosomes under VSG silencing also reduce the rate of VSG turnover to further conserve coat density. Our data indicate that trypanosomes do not regulate VSG turnover according to VSG protein abundance, nor was there any effect on the post-Golgi transport of soluble or GPI-anchored secretory cargo. However, the surface morphology of silenced cells was altered from a typically rugose topology to a smoother profile, consistent with reduced overall membrane trafficking to the cell surface.

**IMPORTANCE** African trypanosomes evade the host immune system by altering the expression of variant surface glycoproteins (VSGs) in a process called antigenic variation. VSG is essential, and when its synthesis is ablated by RNAi silencing, cells enter precytokinesis growth arrest as a means to maintain constant cell surface VSG levels. We have investigated whether arrested cells also alter the rate of natural VSG turnover as a means to conserve the surface coat. This work provides insights into the natural biology of the glycocalyx of this important human and veterinary parasite.

## INTRODUCTION

African trypanosomes (Trypanosoma brucei spp. and other salivarian species) are the causative agents of human African trypanosomiasis (HAT) (African sleeping sickness) and the cattle wasting disease Nagana. Although HAT incidence has declined dramatically in the last decade (1, 2), Nagana still poses a huge socioeconomic burden to sub-Saharan Africa, where millions of livestock are at risk for developing the disease. Furthermore, these livestock are also a well-defined reservoir for HAT-causing trypanosomatids, complicating the goal of human disease elimination. The parasite is digenetic and so alternates between the tsetse fly vector (genus *Glossina*) and the mammalian host, where it can be found in the circulation, skin, adipose tissue, and central nervous system (3–5). Each bloodstream parasite is covered by a dense monolayer of a single homodimeric variant surface glycoprotein (VSG), attached to the membrane by glycosylphosphatidylinositol (GPI) anchors (6). VSG makes up >90% of the cell surface proteome and so shields underlying invariant membrane proteins from host immune recognition. The extracellular parasite, which has upwards of 2,000 *VSG* genes, evades the host immune system by a complex process of antigenic variation involving the periodic “switching” of its VSG coat (6, 7). In this process, monoallelic expression in any single cell is switched from one *VSG* gene to another, and the old coat is replaced by a combination of cell division and innate turnover (8).

Under normal growth conditions, VSG is very stable, with a half-life (*t*_1/2_) of ~26 h (9–11). Turnover is a bimodal process involving both GPI hydrolysis and direct shedding of VSG with intact GPI anchors (11). VSG is essential for cell viability; RNA interference (RNAi) silencing of expression leads to rapid precytokinesis cell cycle arrest and the global cessation of translation (12, 13). Treatment of cells with VSG-specific translation-blocking RNA morpholino oligonucleotides causes an identical phenotype, indicating that premitotic arrest likely occurs by monitoring VSG protein synthesis and/or abundance (14). It is thought that the VSG cell cycle checkpoint is meant to conserve VSG density on the cell surface in order to protect the underlying membrane and proteins from immunological attack. Blocking VSG production does not affect lysosomal trafficking of the endogenous reporter *Tb*CatL, indicating that sequential endoplasmic reticulum (ER)-to-Golgi and then Golgi-to-lysosome trafficking are kinetically unimpaired (14). However, the numbers of coupled ER exit sites (ERESs) and Golgi stacks per cell decrease, and there is a notable distortion of the ER and swelling of *trans*-Golgi cisternae. These data suggest that a reduced secretory flux of VSG, the major secretory cargo, impacts the morphology and maintenance of ER and Golgi structures.

## RESULTS AND DISCUSSION

**FIG 1:**
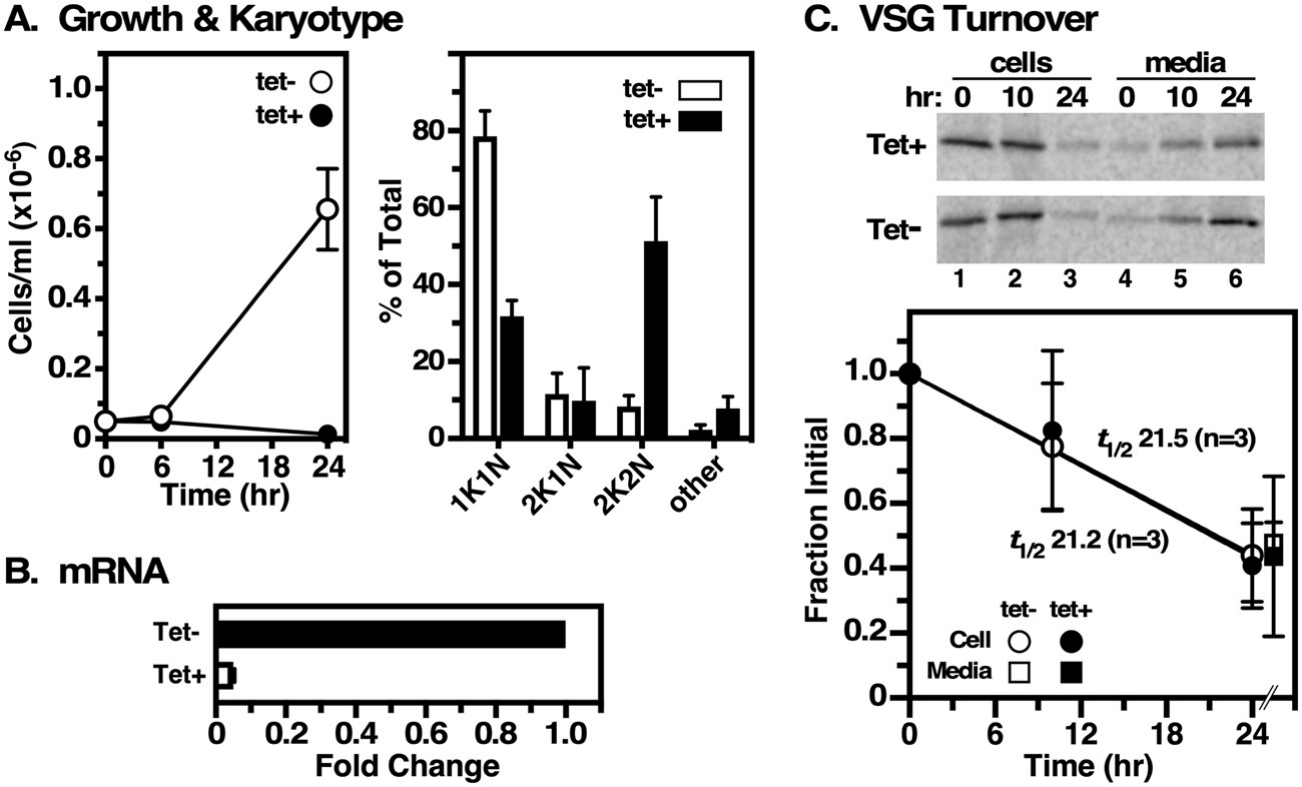
(A, left) Growth curves of VSG RNAi cells without (tet−) and with (tet+) tetracycline induction (means ± standard deviations) (*n* = 3). Cells were seeded at 5 × 10^4^ cells/mL and counted at the indicated time points. (Right) Karyotype analysis of cells without or with induction for 8 h (open versus closed bars) and labeled with 4′,6-diamidino-2-phenylindole (DAPI) (means ± standard deviations) (*n* = 4). (B) The extent of VSG mRNA knockdown was assayed by real-time quantitative reverse transcription-PCR (qRT-PCR) at 8 h. Values are normalized to those for uninduced controls (means ± standard deviations) (*n* = 3 biological replicates). (C) VSG RNAi cells were pretreated without or with induction (Tet−/Tet+) (2 h) prior to experimentation. Pulse (15 min)-chase (24 h) radiolabeling was conducted without or with tetracycline. Cell and medium fractions were immunoprecipitated at the indicated time points and quantified by SDS-PAGE and phosphorimaging (means ± standard deviations) (*n* = 3). A representative image is presented.

We sought to determine if the VSG-associated cell cycle checkpoint also results in the downregulation of VSG turnover, further conserving surface coat density while VSG protein is not being synthesized. To this end, we recreated the RNAi cell line described previously (12, 13). Consistent with those reports, VSG RNAi had an immediate growth effect within the measured time period, with postmitotic accumulation of arrested cells containing two kinetoplasts (k) and two nuclei (n) after 8 h (>50%) (Fig. 1A). Quantification indicated ~95% knockdown of VSG mRNA relative to uninduced controls at 8 h of induction. We then performed our standard 24-h pulse-chase analysis of VSG turnover (11) (Fig. 1C). Prior to radiolabeling, cells were cultured for 2 h without (control) or with tetracycline to initiate VSG silencing, which was then maintained throughout the chase period. At this time point, VSG synthesis is still 90% of that of the control; at 8 h, it is 30% (14). As seen previously, intact VSG is shed into the medium over the 24-h chase period. In each case, cells displayed essentially identical turnover rates (*t*_1/2_ of ~21 h) that were comparable to those previously observed in wild-type (WT) cells (11). These results indicate that VSG turnover remains the same in the absence of VSG synthesis, and thus, turnover is not regulated according to the amount of VSG protein present in the cell.

As noted above, RNAi silencing of VSG synthesis causes rapid translational and precytokinesis cell cycle arrest, with a global arrest of protein synthesis, to conserve cell surface VSG density (12–14). One sequela of this phenomenon is that the morphology of the early secretory pathway is altered—the average numbers of ERESs and associated Golgi stacks decrease on a per-cell basis—but counterintuitively, *trans*-Golgi cisternae are dilated. The latter observation was interpreted to indicate that anterograde vesicular transport from the Golgi stacks is reduced in the absence of VSG cargo (14). Since Golgi-to-lysosome transport of *Tb*CatL is normal under VSG ablation, and no disruption of lysosomal morphology was reported (14), it must be the secretory arm of post-Golgi sorting that is impaired.

**FIG 2:**
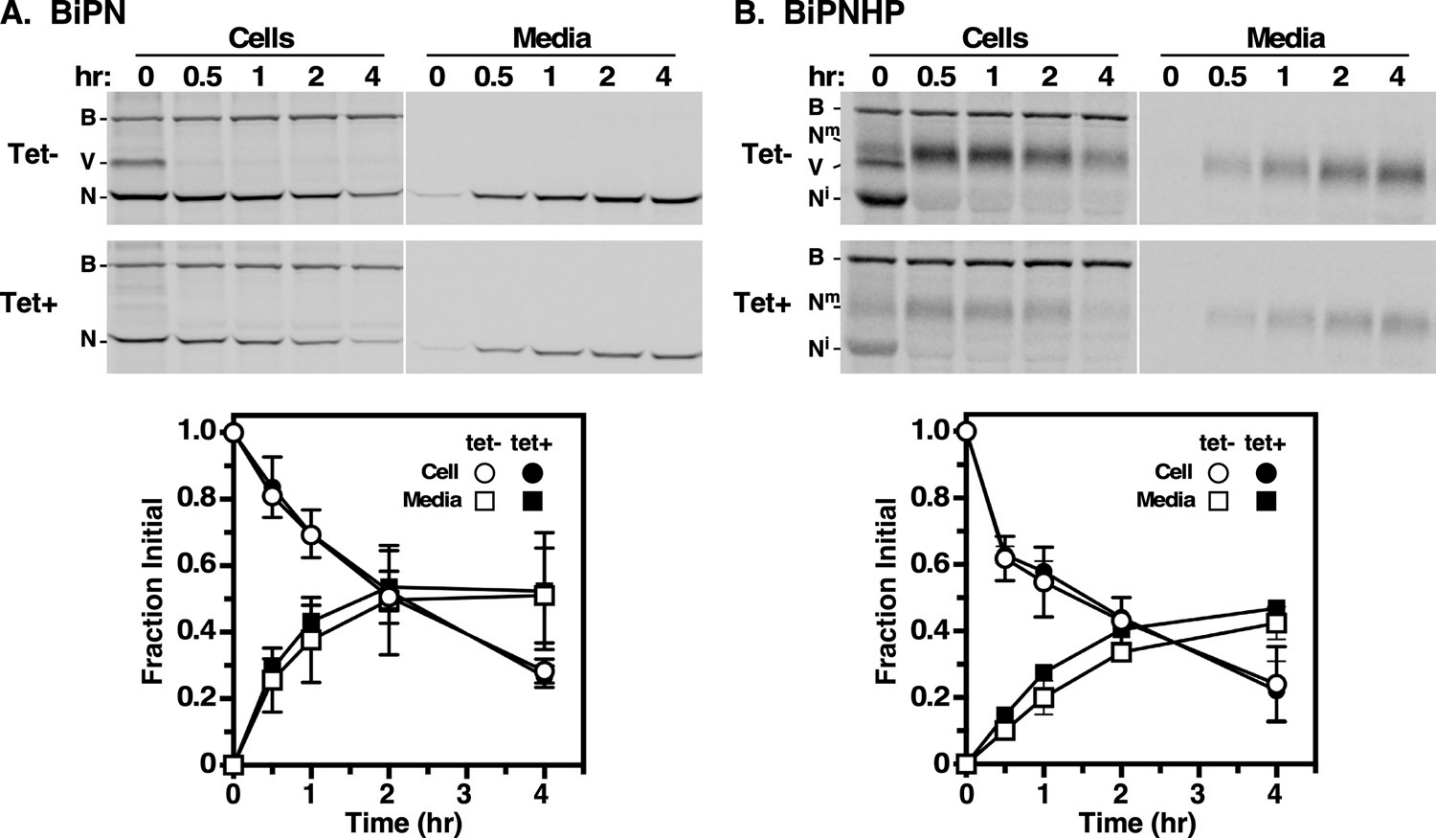
Matched BiPN (A) and BiPNHP (B) reporter cell lines were treated (8 h) without (tet−) (open symbols) or with (tet+) (closed symbols) induction of VSG dsRNAi synthesis. Cells were then pulse (15 min)-chase (4 h) radiolabeled, and reporter transport and processing were analyzed by immunoprecipitation/SDS-PAGE. Representative phosphorimages of cell and medium fractions are presented. Matched tet+ and tet− cell lines were image processed identically. White lines indicate lanes that were removed after processing to enhance presentation. Note that total incorporation is lower in tet+ cells as VSG silencing leads to global translational repression (13). Loss from cells and appearance in the medium were quantified as a fraction of the value for the initial cell-associated reporter (means ± standard deviations) (*n* = 3). Mobilities are indicated as follows: B, native BiP; V, VSG; N, BiPN; N^i^, immature BiPNHP; N^m^, mature BiPNHP.

To test this, we constitutively expressed secretory reporters, soluble BiPN and GPI-anchored BiPNHP, in the VSG RNAi cell line. These matched reporters are based on the globular N-terminal ATPase domain of the ER molecular chaperone BiP. Soluble BiPN is transported to the flagellar pocket and then secreted to the external milieu (15, 16). BiPNHP has a GPI attachment peptide fused to the C terminus. It is rapidly transported to the cell surface and then shed into the medium with an intact GPI anchor (16, 17). *En route*, it receives extensive processing of the GPI glycan core, leading to a substantial increase in the overall molecular mass (18). These matched reporter cell lines were subjected to pulse-chase metabolic radiolabeling and transport, followed by immunoprecipitation. In control cells, soluble BiPN was transported and secreted into the medium with typical kinetics (Fig. 2A). Endogenous full-length BiP remained cell associated and served as a loading control. Newly synthesized VSG, which transiently associates with native BiP during the folding process (19), was also seen at the 0-h time point (*T*_0_). Silencing eliminated this early BiP-associated VSG but had no effect on the transport and secretion of BiPN. Identical analyses were performed with the BiPNHP cell line (Fig. 2B). Again, full-length endogenous BiP remained cell associated, and newly synthesized VSG was transiently detected in pulldowns in control cells. As expected, newly synthesized BiPNHP was initially detected as a discrete smaller immature species (N^i^). During intracellular trafficking, it was converted to the larger mature form (N^m^) and was ultimately shed into the medium with typical kinetics. Silencing eliminated BiP-associated VSG at the initial time point but had no effect on reporter transport and shedding. These results indicate that the secretory arm of post-Golgi trafficking is not impaired by VSG silencing, as the transport and secretion/shedding of the matched reporters were unaffected.

**FIG 3:**
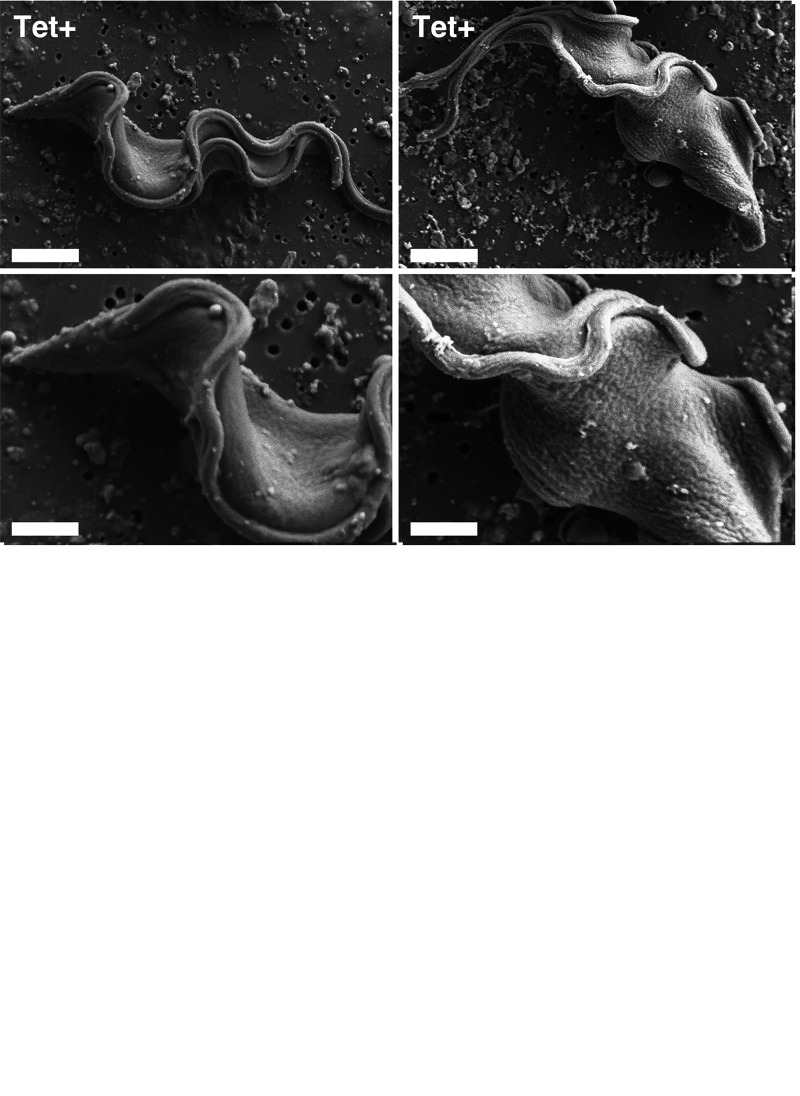
Scanning electron microscopy images of control (Tet−) and silenced (Tet+) cells after 8 h of culture. Matched low (top)- and high (bottom)-magnification images are presented. Bars, 2 μm (top) and 1 μm (bottom). White and black arrowheads indicate old and new flagella, respectively (low-magnification control only).

Despite the lack of an apparent effect on anterograde trafficking, the observed dilation of *trans*-Golgi cisternae (14) suggests that subsequent alterations to cell surface topography might be expected. When examined by scanning electron microscopy (SEM), precytokinesis control cells had a typical rugose, i.e., corrugated, cell surface morphology (Fig. 3), as we have noted previously (11, 20). However, silenced cell cycle-arrested cells had markedly decreased corrugation and increased smoothing of the cell surface after 8 h of induction. VSG is the overwhelmingly major secretory cargo of bloodstream-form (BSF) trypanosomes, and its loss seemingly decreases vesicular traffic departing the Golgi stacks for the cell surface, resulting in cisternal dilation. Nevertheless, enough remaining capacity is apparently retained to allow the unaffected transport of other extracellular secretory cargo; i.e., the amount of departing cargo vesicles mirrors the flux of cargo awaiting transport. Endocytosis is not affected by VSG silencing (21), nor is recycling, since at the normal rate of endocytosis, the entire cell surface would be internalized and consumed in 12 min (22). Thus, the decrease in the total vesicular membrane arriving at the cell surface results in net reductions in the total surface/volume ratio, as noted in the original work on VSG silencing (12), and, hence, in rugosity. This in turn contributes to maintaining critical VSG density in the absence of ongoing biosynthesis.

**FIG 4:**
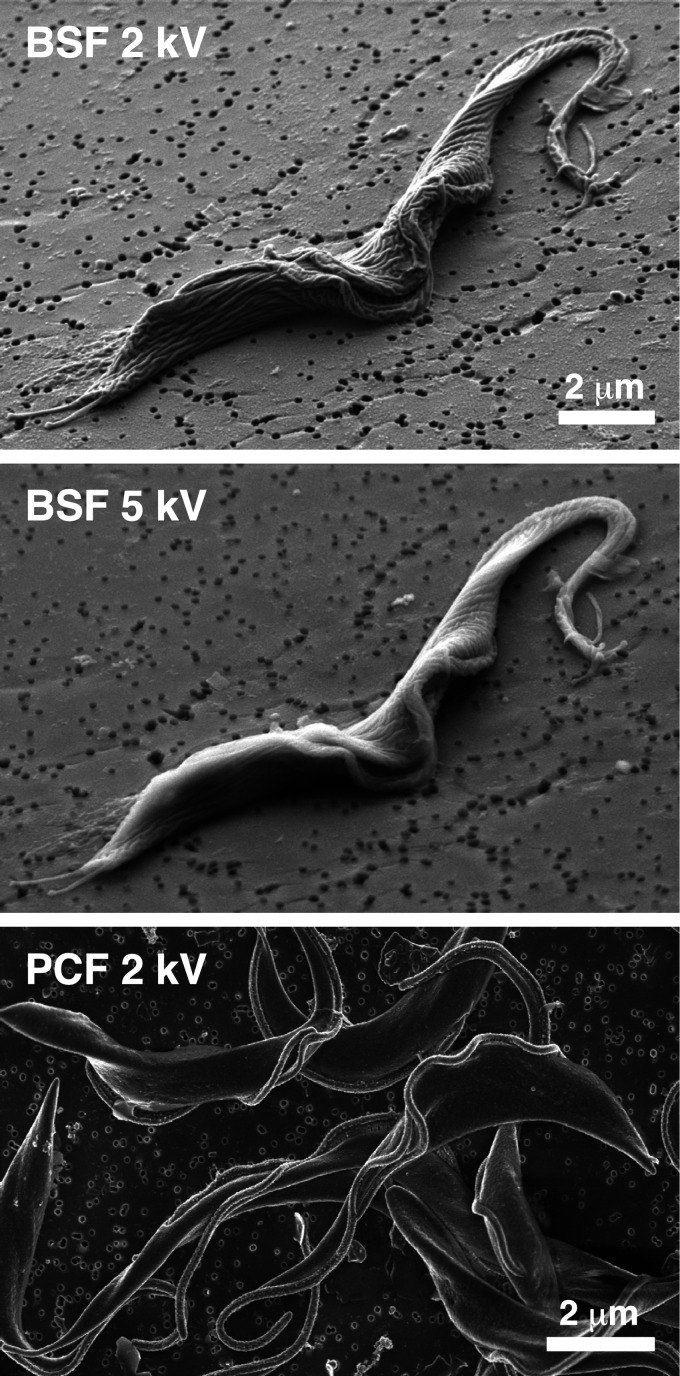
SEM of normal BSF (top and middle) and procyclic form (PCF, bottom) trypanosomes at different voltages. BSF images were acquired at an oblique angle; the PCF image was acquired vertically.

It should be noted that in the original description of VSG RNAi (12), SEM images were presented that did not show the rugose morphology that we typically observe in normal cells (11, 20). This may be due in part to methodology: final dehydration by critical point drying (12) versus hexamethyldisilazane (us). However, another possible factor is the voltage of image acquisition. Increased voltage can lead to image blurring (smoothing), as is shown with a modest increase of just 2 to 5 kV (Fig. 4). Sheader et al. (12) do not report the voltage used, but if it was in excess of 2 kV, it is possible that initial rugosity in control cells was missed. Whatever the explanation for these differences, the rugosity that we see in control versus silenced cells is clearly the result of biological conditions and not sample preparation. Likewise, we see little rugosity in normal procyclic cells (Fig. 4), again arguing that our images of normal bloodstream cells are not a methodological artifact but rather represent the native biological situation.

Our findings may shed light on three phenomena associated with the ablation of VSG synthesis, as reported previously by Cheung et al. (21). First, while silenced cells can persist for days *in vitro* in an arrested state, they are rapidly cleared over 10 to 12 h from the blood of immunologically naive mice. It is likely that in this situation, the continuing loss of VSG (and the lack of VSG antigenic switching) renders the parasite increasingly susceptible to innate arms of the host immune system, such as opsonization by the alternate complement pathway, leading to lysis and phagocytosis by macrophages. Second, VSG-depleted cells have markedly increased directional motility (less tumbling), which is attributed to increased cellular rigidity. We suggest that this loss of flexibility may be a natural consequence of the “contraction” of the total surface area that we observe by SEM. Finally, silenced cells have a reduced rate of clearance of surface-bound anti-VSG antibody. This was also attributed to the loss of flexibility and motility, but another contributing factor may be changes to the lipid composition of the plasma membrane due to reduced anterograde vesicular trafficking in the presence of ongoing membrane recycling/turnover.

## MATERIALS AND METHODS

### Cell lines and culture.

All work was carried out with cultured log-phase BSF cells of the commonly used tetracycline-responsive single-marker (SM) Lister 427 strain of T. brucei *brucei* (variant antigenic type MITat1.2 expressing VSG221) (23) grown in HMI9 medium at 37°C with 5% CO_2_ (24). VSG RNAi cells were made using plasmid pHNES221ES_BLAST (12) to lock the BES1 expression site and the VSG 221 RNAi construct and pLEW100.V5x.PEX11.SL221RNAi (13), both obtained from Gloria Rudenko, Imperial College. Induction of double-stranded RNA (dsRNA) synthesis was achieved with 1 μg/mL of tetracycline.

### Quantitative real-time PCR.

The levels of mRNA knockdown of VSG 221 RNAi cells were quantified using VSG specific primers (forward primer [FP] 5′-CGCTGAAAGCCAACAACAAG-3′ and reverse primer [RP] 5′-CCGCATCGTTATGCCATTTG-3′). RNA isolation, DNase treatment, cDNA synthesis, and quantitative PCR were performed as described previously (20). TbZFP3 was used as a reference gene.

### VSG turnover assay.

Log-phase cultured BSF trypanosomes were RNAi silenced under tetracycline induction for 2 h prior to pulse-chase analysis. Following induction, cells were washed in HBS, and subsequent analysis was performed as described previously (11), with and without tetracycline to maintain silencing. Briefly, cells were pulse radiolabeled (15 min) with [^35^S]methionine-cysteine (Perkin-Elmer, Waltham, MA), washed, resuspended in HMI9 medium with or without tetracycline, and incubated for the indicated times. Samples were lysed in radioimmunoprecipitation assay (RIPA) buffer (50 mM Tris HCl [pH 8.0], 100 mM NaCl, 1% NP-40, 0.5% deoxycholate, 0.1% SDS), and labeled VSG 221 polypeptides were subsequently immunoprecipitated. Pulse and chase times are indicated in the figure legends. All immunoprecipitates were fractionated by 12% SDS-PAGE and imaged using a Molecular Dynamics Typhoon FLA 9000 system.

### BiPN secretion assay.

The secretory reporters BiPN (BiPN:AVRG [19]) and BiPNHP (17, 18) (also known as BiPN:GPI [16]) were constitutively expressed in the VSG RNAi cell line. Analyses of reporter transport and secretion/shedding were performed by pulse-chase (15 min/4 h) metabolic radiolabeling and immunoprecipitation as described previously (16).

### Scanning electron microscopy.

Scanning electron microscopy (SEM) analysis was performed as described previously (20). Briefly, trypanosomes were fixed at 4°C in HMI9 growth medium containing 2.5% glutaraldehyde for 30 min and then captured on 0.2-μm-pore-size polycarbonate filters. The filter was washed, dehydrated, and carbon coated in a high-vacuum evaporator. Filters were inspected using a Hitachi SU70 field emission scanning electron microscope operated at 2.0 keV.

### Data analyses.

ImageJ (http://imagej.nih.gov/ij/) was used to quantify data from phosphorimager assays and fluorescence blot scans. For quantitative analysis of band intensities, signals from each lane were subtracted from the signal of the equivalent unlabeled areas of that lane. In the case of the VSG turnover assay, the zero supernatant signal was subtracted from all subsequent supernatant signals. Data analyses were performed with Prism6 software (GraphPad Software, Inc., San Diego, CA). Biological replicates were obtained as indicated.
